# Identification of salmoniformes aquaculture conditions to increase creatine and anserine levels using multiomics dataset and nonnumerical information

**DOI:** 10.3389/fmicb.2022.991819

**Published:** 2022-10-28

**Authors:** Hideaki Shima, Izumi Murata, Wei Feifei, Kenji Sakata, Daiki Yokoyama, Jun Kikuchi

**Affiliations:** ^1^RIKEN Center for Sustainable Resource Science, Yokohama, Japan; ^2^Graduate School of Medical Life Science, Yokohama City University, Yokohama, Japan; ^3^Graduate School of Bioagriculuture Sciences, Nagoya University, Nagoya, Japan

**Keywords:** anserine, aquaculture, creatine, intestinal microbiota, metabolites, salmoniformes

## Abstract

Aquaculture is attracting attention as a sustainable protein source. Salmoniformes, which are generally called salmon, are consumed in large quantities worldwide and are popularly used for aquaculture. In this study, the relationship between muscle metabolites, intestinal microbiota, and nonnumerical information about the ecology of salmoniformes was investigated to improve the efficiency of aquaculture using nuclear magnetic resonance and next-generation sequencing with bioinformatics approach. It was revealed that salmoniformes are rich in anserine and creatine, which are useful for human health care, along with collagen and lipids. The important factors in increasing these useful substances and manage the environment of salmoniformes aquaculture should be noted.

## Introduction

With the ever-growing population, food security, and sustainable protein supply have become urgent issues. Aquaculture is expected to play a critical role as a sustainable global food supply because it has smaller carbon footprints using land and water than other cultures (Poore and Nemecek, 2018; Pradeepkiran, 2019; Henriksson et al., 2021). Additionally, consumers also consider the taste, texture, and safety of farmed fish (Hardy and Lee, 2010). However, various fish must be properly bred, and some are also difficult to breed (Kagawa et al., 2005). Therefore, it is necessary to investigate the fish ecology, phenotype, optimal environment for farming, and their relationships.

In recent years, nuclear magnetic resonance (NMR) and mass spectrometry have made it possible to analyze comprehensive substances like metabolites. Next-generation sequencing (NGS) has made it possible to analyze bacteria that were difficult to culture using nucleic acids. Furthermore, advances in informatics and computer performance are able to mine useful information from datasets, and new calculation methods are still being proposed (Wei et al., 2021; Shima et al., 2022).

With the advances in these technologies, the role of symbiotic bacteria has become known. Their roles are diverse, which include providing nutrients to the host by degrading food residues in the gut, developing immunity, and affecting mind and behavior (Osawa et al., 1995; Forsythe et al., 2010; Ray et al., 2012; Chaix et al., 2014; Ohno, 2020). In other words, if symbiotic bacteria can be controlled, it is considered possible to modify the appropriate host conditions. The effects of diet or culture on intestinal microbes have also been researched, and feeding useful bacteria or food to the host include the concepts of probiotics and prebiotics (Hehemann et al., 2010; Pandey et al., 2015; Shima et al., 2017).

In this study, we focused on salmoniformes, which include *Oncorhynchus*, *Salmo*, and *Salvelinus*, which are popular fish species in land-based and sea-based aquaculture. The characteristics of salmoniformes muscle metabolites and intestinal microbes and those of other fishes were determined, and association rules among intestinal microbes, muscle metabolites, and nonnumeric information on salmoniformes were investigated by i-means, which has two machine learning parts developed by our group for mining meaningful variables in multiple datasets. We report that bacterial and metabolite clustering information are associated with nonnumerical fish information, and some bacteria affect the concentrations of muscle metabolites in salmoniformes.

## Materials and methods

### Sample collection and preparation

A total of 121 salmoniformes samples were collected from Japan river, lake, pond, and sea or were received from a commercial grower [*Oncorhynchus mykiss* = 48 (landlocked), *O. masou* = 10 (catadromous), 19 (landlocked), *O. nerka* = 13 (landlocked), *O. kisutch* = 22, *Salvelinus malma* = 3, *Salvelinus leucomaenis* = 2, *Salmo trutta* = 4]. The samples were stored at −30°C until dissected. Frozen samples were thawed under running water. The length from head to tail and weight of thawed samples were measured. Then, their intestinal contents were separated, collected to a sample tube, and freeze-dried. The fish were halved, and their bones were removed. Half of the muscles was peeled off and minced, while the other half were used for physical tests. The minced muscles were collected to a sample tube and freeze-dried. For NMR, 110 salmoniformes samples were used, and for NGS, 52 salmoniformes samples were used included pooled data. Both NMR and NGS were performed in 41 samples. For collagen and lipid studies, 89 salmoniformes samples were used.

### NMR data

Freeze-dried minced muscles were powdered and 18 mg of the powdered sample was suspended in 600 μl of KPi buffer containing 90% deuterium oxide and 1 mM sodium 2,2-dimethyl-2-silapentane-5-sulfonate (DSS). The samples were then kept at 65°C for 15 min and centrifuged at 17,800 G for 5 min. Two-dimensional *J*-resolved (2D *J*-RES) NMR spectra were acquired at 298 K using a Bruker AVANCE II 700 spectrometer equipped with a ^1^H inverse triple-resonance cryogenically cooled probe with Z-axis gradients (Bruker BioSpin GmbH, Rheinstetten, Germany). In brief, 2D *J*-RES NMR spectra were acquired using the standard Bruker pulse program jregpprqf, with 16,384(F2) and 32(F1) data points, and data were collected from 16 transient and 16 dummy scans.

### Physical test

Half of the fish muscles were thawed at 25°C ± 5°C and cut off (width = 10 mm, thickness = 5 mm, length = 50 mm). The shard muscles were tested for cutting strength by EZ-L (Shimadzu Co. Ltd., Kyoto, Japan). The machine setting was 40 N (force), 5 mm (stroke), and 2 mm min^−1^. Distance was measured over time at least thrice. The force data were used to make a logarithmic approximation curve, and A and B values were obtained according to a previous report (Wei et al., 2021).

### Fish gut microbe analysis (NGS data)

Intestinal microbial DNA was extracted based on a previous report (Date et al., 2012). In brief, DNA was extracted from the intestinal contents using the ethanol precipitation method and then amplified *via* PCR using the universal primers 954F and 1369R (Eurofins Genomics K.K., Tokyo, Japan), dNTPs, and ExTaq (Takara Bio Inc., Shiga, Japan). These primers were designed to amplify regions V6–V8 of the 16S rRNA coding region. The PCR procedure conditions were set as follows: 95°C for 4 min; 35 cycles of 95°C for 4 min, 95°C for 30 s, 45°C for 30 s, and 72°C for 1 min; followed by 72°C for 10 min. The PCR products were purified using AMpure (Beckman Coulter, Inc., Brea, United States), and DNA was quantified using the Qubit Fluorometer (Thermo Fisher Scientific, Waltham, United States). Each sample was diluted to the same concentration and pooled into one library. The pooled library and PhiX Control v3 (Illumina, Inc., San Diego, United States) were denatured with 0.1 M NaOH, and the library was diluted to a final concentration of 3.5 pM with the HT1 hybridization buffer spiked with the PhiX solution. The final solution was analyzed using the Miseq Reagent Kit V2 (Illumina, Inc., San Diego, United States) on Miseq (Illumina, Inc., San Diego, United States). An average of 2,393 sequences were obtained for 46 of the 121 salmoniformes samples. Sequence data in FASTQ format were processed using QIIME. The forward and backward reads were joined, and the chimeras were filtered out. An operational taxonomic unit was defined at 97% similarity and assigned taxonomic references based on the RDP database.

### Data processing and analysis

#### Data processing

NMR data were normalized according to internal standard DSS, and 233 regions of interest were defined by Revolution R Open software.^1^ Peaks were annotated by our metabolite database. NMR signals and NGS data were processed to get the composition ratio to the sum of the measurement. For association analysis by *a priori*, A and B values, fish length and weight, and lipid and collagen data were ranked within the strain, and the top and bottom quarters of the ranking were converted as “high” and “low,” respectively. Additionally, NMR and NGS data were converted according to the ranking within an individual.

#### Pooled data

We used the pooled fish datasets available in our laboratory as the control group for salmoniformes. We used 1,858 pooled datasets for NMR and 204 pooled datasets for NGS. Each pooled dataset had 21 orders of taxonomy. We used 799 samples datasets for collagen and lipid studies.

#### Computational analysis

We used open software R (see text footnote 1) and package “arules” for association analysis, “randomForest” for classification, and i-means for clustering, which was a combination analysis based on K-means and random forest (Shima et al., 2022). Class information computed using i-means was added to the NMR and NGS datasets of salmoniformes. Furthermore, habitat information (river, lake or pond, name of spot) and fish species were also added. We selected association rules over 1 lift value. The association rule network was depicted using Gephi, an open-source software.^2^

## Results

### Comparison of salmoniformes with other fishes

**Figure 1:**
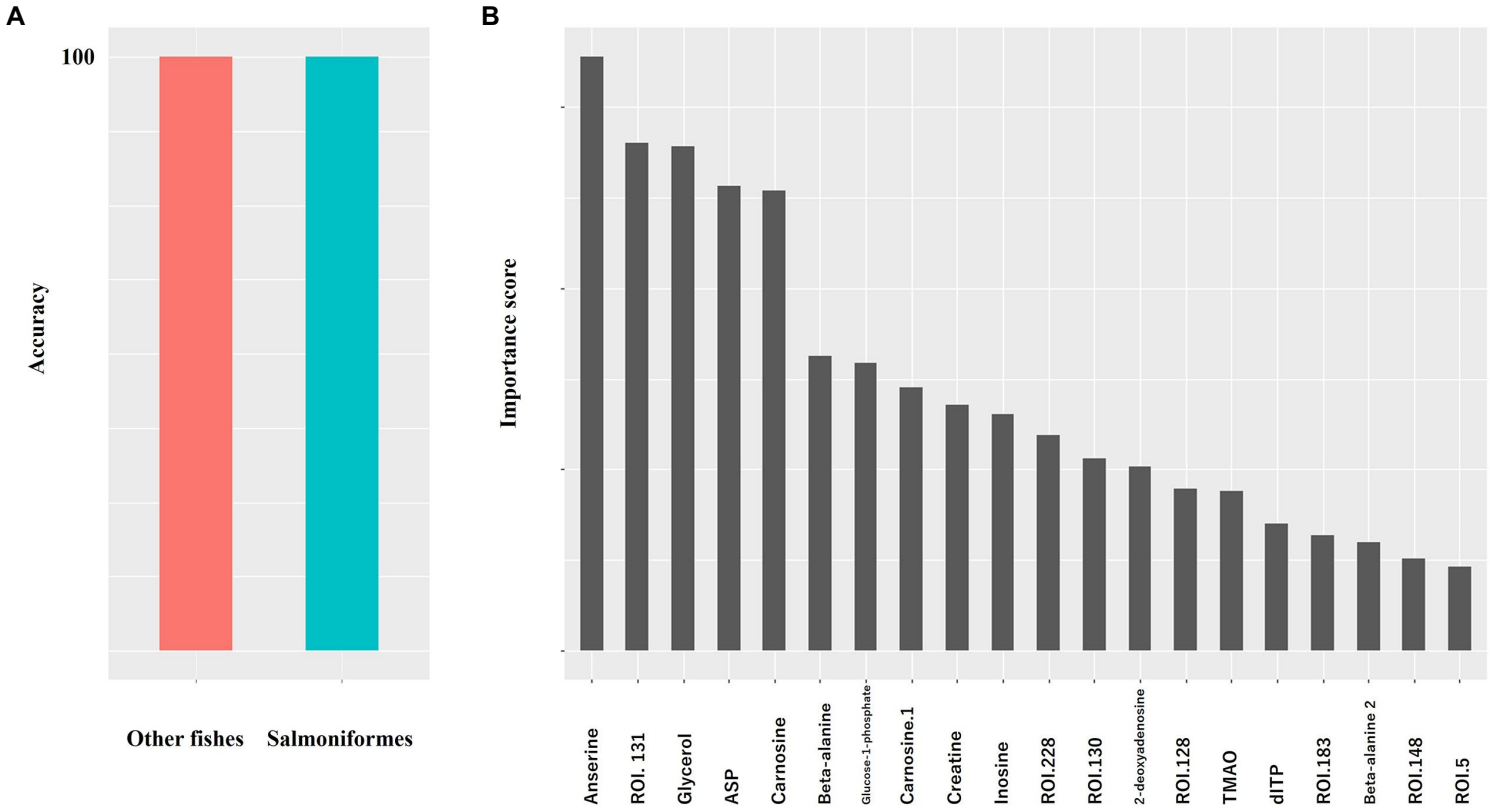
Random forest results of metabolite data to classify other fishes and salmoniformes. We used pooled data for other fishes (other fishes: *n* = 1858, salmoniforems: *n* = 110). **(A)** The accuracy of classification was determined using random forest. Values are the averages of five measurements; however, the values were not dispersed. **(B)** In NMR, a higher score indicates a higher importance to classify. The importance scores are shown in Table 1.

**Figure 2:**
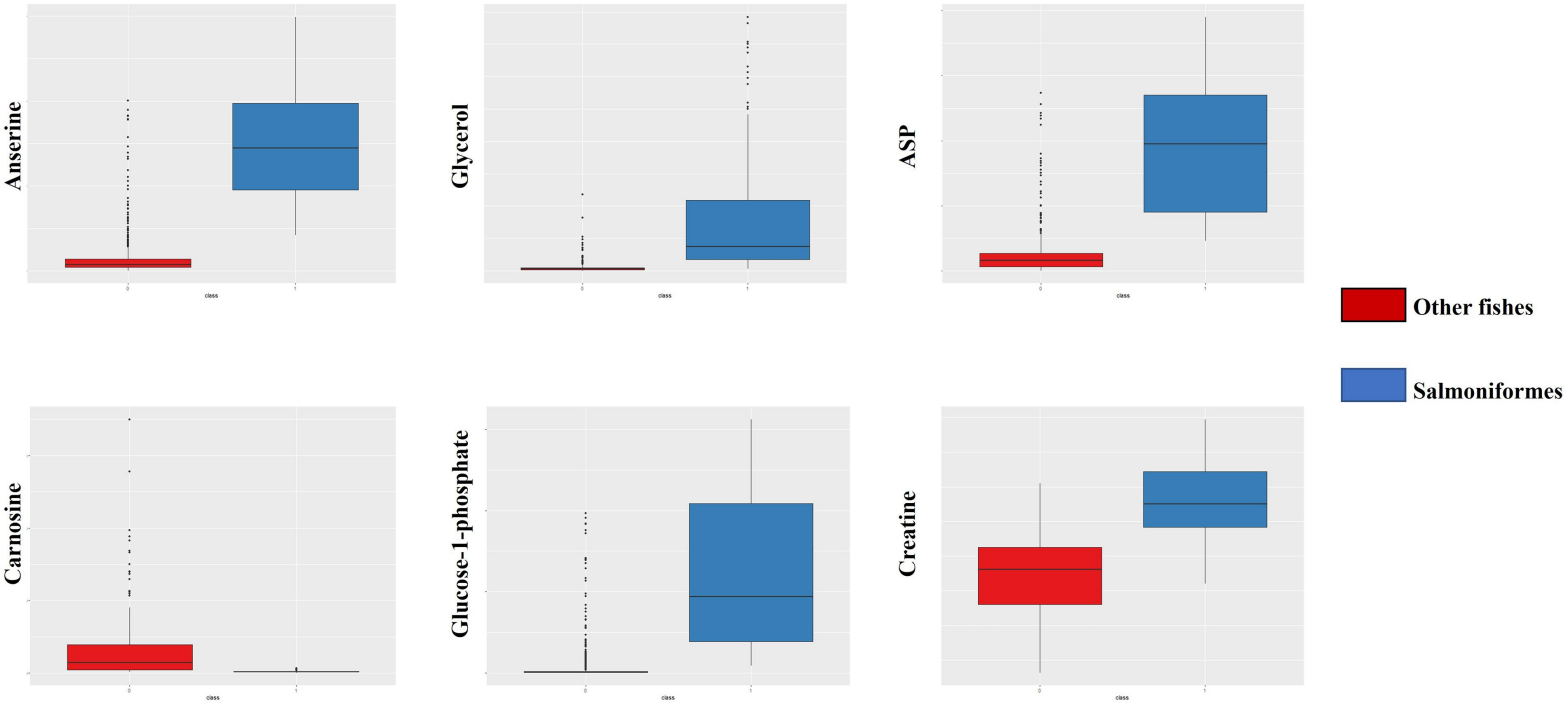
Box plot of important factors of NMR data. From the upper left, the panel indicates anserine, glycerol, and aspartic acid. From the lower left, the panel indicates carnosine, glucose-1-phosphate, and creatine. The red box indicates other fish data, and the blue box indicates salmoniformes. It can be seen that salmoniformes had higher scores, except for carnosine. The vertical axis indicates the composition ratio to the total amount of intensity. In the NMR panels, the number of other fishes is 1858 and the number of salmoniformes is 110.

First, we compared the data (NMR: *n* = 1,858, NGS: *n* = 204) pooled in our laboratory with the data from a salmoniformes study to clarify the differences between salmoniformes and other fish species based on random forest. As a result, the accuracy of using the NMR dataset to classify other fishes and salmoniformes was 100% (Figure 1A), and important variances to classify NMR data set were extracted (Figure 1B; Table 1). On the other hand, although the accuracy of the NGS dataset was slightly lower (Supplementary Figure S1A), it was possible to classify and extract important factors (Supplementary Figure S1B; Supplementary Table S1). Boxplots were created to visualize some of these highly important factors to classify other fish species and salmoniformes (Figure 2; Supplementary Figure S2). In our pooled data, although anserine was high in the muscles of some fish species, it was low in many other fish species (21 orders of taxonomy), except for salmoniformes. It was found to be abundant in the muscles of salmoniformes. Additionally, it was also suggested that glycerol, aspartic acid, glucose 1-phosphate, and creatine tended to be abundant in salmoniformes, while carnosine was found to be less abundant. Based on NGS results on actinobacteria, it was less in salmoniformes, while it was not significantly different in other phyla. High-molecular weight lipids and collagen were also checked and compared among other fish and salmoniformes; it was found that both lipids and collagen were higher in salmoniformes (Supplementary Figure S3).

**Table 1:** NMR importance ranking for separating salmoniformes and other fish species computed using the random forest method.

	Name or ROI number	Score
1	Anserine	16.37
2	ROI.131	14.02
3	Glycerol	13.92
4	Asp	12.82
5	L.Carnosine	12.69
6	Beta.alanine.2	8.13
7	Glucose.1.phosphate	7.93
8	L.Carnosine.1	7.26
9	Creatine	6.79
10	Inosine	6.51
11	ROI.228	5.95
12	ROI.130	5.29
13	2.deoxyadenosine	5.09
14	ROI.128	4.47
15	TMAO	4.40
16	dITP	3.49
17	ROI.196	3.18
18	Beta.alanine.1	2.97
19	ROI.148	2.55
20	ROI.5	2.33

The score indicates gini impurity, a higher value of which indicates a more important variable for classification. Signals identified in our database are named. Unidentified signals are marked with ROI numbers.

### Classification of metabolites and intestinal microbes in salmoniformes

**Figure 3:**
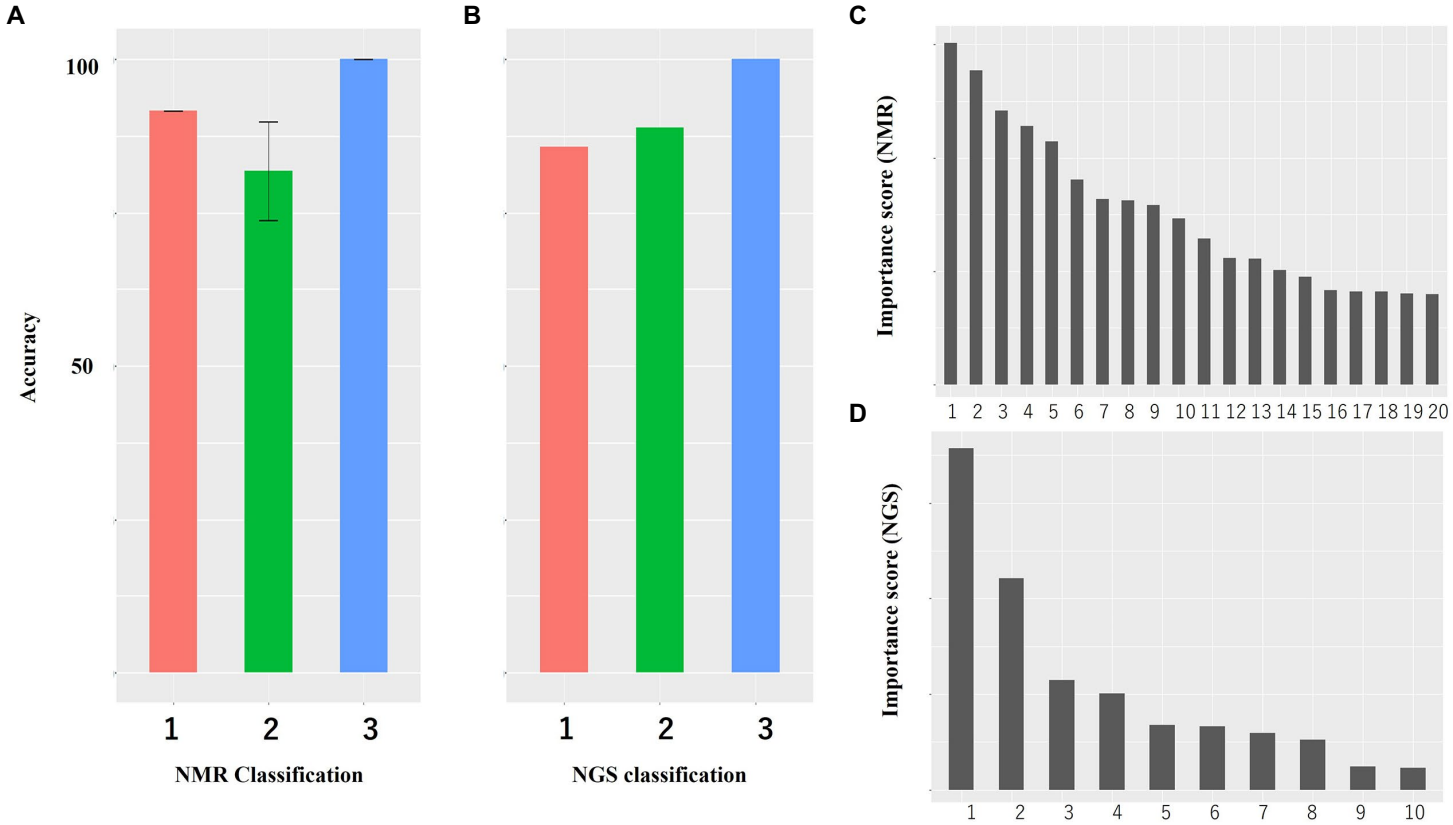
Accuracy of classification of NMR data and NGS data and histogram of importance computed using i-means. **(A)** Both NMR and **(B)** NGS data are highly accurate. The numerical value is the average value of five measurements. Bars indicate standard deviation, and no bars indicate no variation. **(C,D)** Histogram of important variables of NMR **(C)** and NGS **(D)** computed using i-means. Important factors were extracted. The importance scores are shown in Tables 2, 3. **(A,B)** The numbers indicate the number of classes divided using i-means. The rankings of panels **C,D** are shown in Tables 2, 3, respectively. There were originally four groups for NGS, but one group including one sample was omitted because it was microbiota composition extremely.

**Figure 4:**
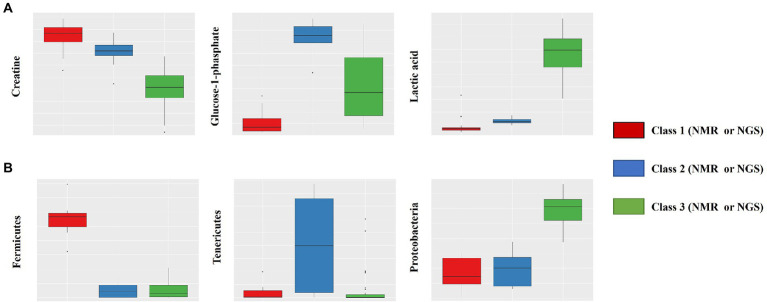
Box plot of important factors extracted using the i-means method. The upper row **(A)** shows the results of NMR data, which includes creatine, glucose-1-phosphate, and lactic acid (from the left). The lower row **(B)** shows the results of NGS data, which includes fermicutes, tenericutes, and proteobacteria (from the left). The vertical axis indicates the composition ratio to the total amount. The number of NMR data sets is 110, and the number of NGS datasets is 51 (excluding one). In the NMR panels, the numbers for classes 1, 2, and 3 are 24, 11, and 75, respectively. In the NGS panels, the numbers for classes 1, 2, and 3 are 7, 9, and 35, respectively.

Intrasalmoniformes classification of metabolites and microbes was performed using i-means, which is based on two machine learning methods, i.e., clustering and classification. i-means is a method for provisional classification using the distance of data and evaluates classification system with random forest (Shima et al., 2022). We tried multiple groupings and examined the appropriate number of groups based on the increase in the number of groups and the decrease in the correct answer rate. Then, we decided that it would be appropriate to divide into three groups for NMR data set and four groups for NGS data set. The metabolites extracted from salmoniformes muscles were divided into three groups (Figure 3A) and important factors for classification were identified (Figure 3C; Table 2). In addition, in intestinal microbes, one sample was extreme and had only the Bacteroides phylum and one additional class, so this sample was omitted; however, NGS data set was divided into four groups and important factors were identified (Figures 3B,D; Table 3). Based on a boxplot made based on extracted important factors, metabolite data could be divided into a group with high creatine concentrations and a group with high lactic acid and glucose 1-phosphate levels (Figure 4A). On the other hand, the bacterial composition was divided into three groups: firmicutes, tenericutes, and proteobacteria (Figure 4B).

**Table 2:** NMR importance ranking for separating intrasalmoniformes computed using the i-means method.

	Name or ROI number	Score
1	Lactate	3.01
2	Lactate.1	2.77
3	N.acetylglutamic.acid.1	2.42
4	ROI.85	2.28
5	L.Carnosine.1	2.14
6	Creatine.143	1.81
7	Ile.1	1.64
8	ROI.144	1.63
9	Asp	1.59
10	Creatine.betaine	1.47
11	ROI.27	1.29
12	ROI.29	1.12
13	ROI.155	1.11
14	ROI.150	1.01
15	Val.1	0.95
16	ROI.2	0.83
17	Anserine	0.82
18	Val	0.82
19	X2.Aminoethyl.dihydrogen.phosphate	0.81
20	Glucose.1.phosphate	0.80

The score indicates gini impurity, a higher value of which indicates a more important variable for classification. Signals identified in our database are named. Unidentified signals are marked with ROI numbers.

**Table 3:** NGS importance ranking for separating intrasalmoniformes computed using the i-means method.

	Bacteria	Score
1	Proteobacteria	7.14
2	Firmicutes	4.42
3	Bacteroidetes	2.29
4	Tenericutes	2.01
5	Fusobacteria	1.36
6	Actinobacteria	1.32
7	Cyanobacteria	1.19
8	Bacteria.Other	1.05
9	Acidobacteria	0.49
10	Planctomycetes	0.46

The score indicates gini impurity, a higher value of which indicates a more important variable for classification. The names of bacterial phyla are listed.

### Identification of the relationship among metabolites, microbes, and nonnumerical information

**Figure 5:**
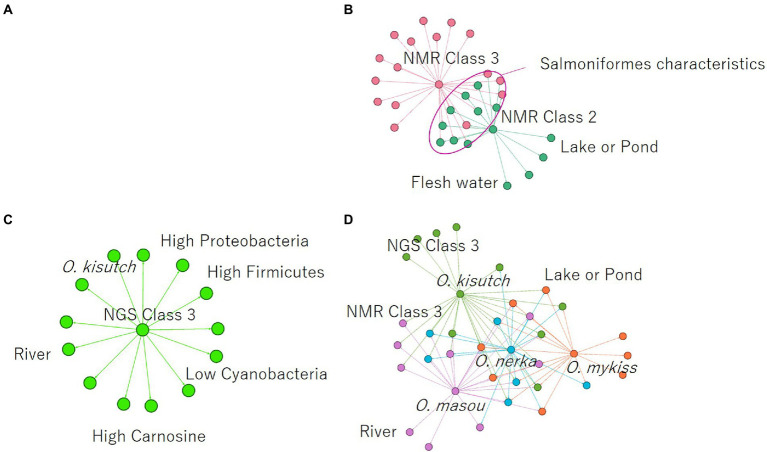
Extraction of association rules using the *a priori* algorithm. **(A)** Rules for all annotated NMR signals, NGS data, and nonnumerical information. **(B)** Association rules centered on NMR class classified using i-means. **(C)** Association rules centered on NGS class classified using i-means. **(D)** Association rules on salmoniformes genus. Color coding indicates modularity automatically calculated by an open software Gephi (https://gephi.org/).

**Figure 6:**
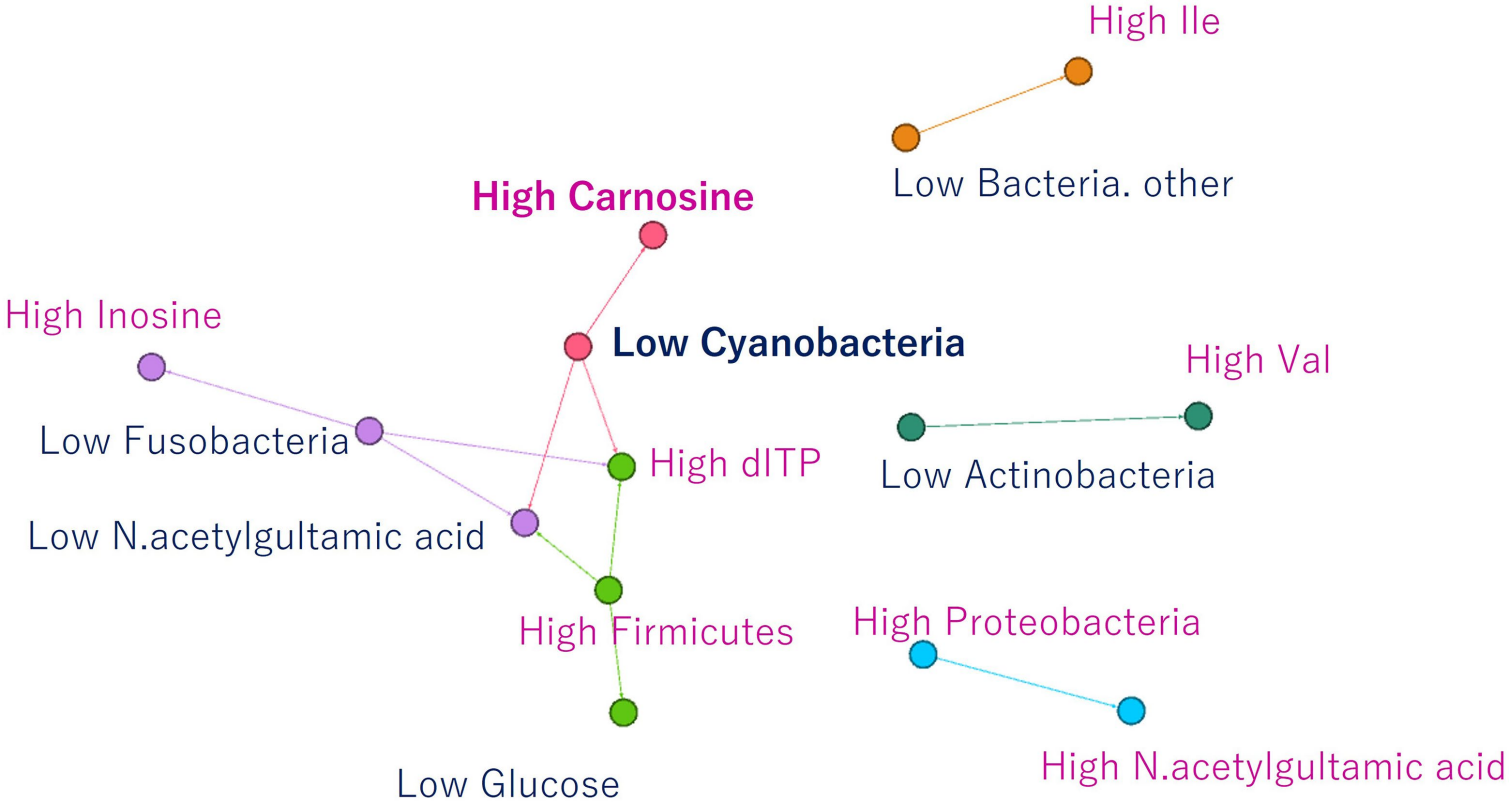
Association rules for NMR signals that increase or decrease due to an increase or decrease in microbes. Three isolated relationships and relationships between three microbes and five metabolites were extracted.

Finally, association analysis (Apriori) was performed to clarify the relationship among metabolites, microbes, and nonnumerical information. We set the top quartile of signal ratios within an individual as “High,” bottom quartile as “Low,” and other values as “NULL.” In addition, nonnumerical information, i-means class information, intraspecific length, weight, muscle hardness, and sample habitat were included. As a result, 29,916 association rules were extracted (Figure 5A). In the class information computed using i-means, one of the three classifications of NMR data set was not found to be correlated to other class, while the remaining two classes shared the characteristics of salmoniformes (Figure 5B). NMR class 2 had four association rules included nonnumerical information “lake or pond” and “flesh water,” with two metabolites while NMR class 3 had 12 characteristics. In the classification of NGS by i-means, no correlation was found between the classes, and 13 pieces of information were found to be involved in only one class (Figure 5C). Additionally, focusing on the association at the genus level of salmoniformes, *O. kisutch* and *O. masou* belonged to NMR class 3 (Figure 5D), while *O. nerka* and *O. mykiss* did not belong to any class. We also extracted the association rules for metabolites that increase or decrease with the microbes to investigate their potential effects on muscles (Figure 6). As a result, some associations were extracted, showing that three single relationships, high proteobacteria – high n acetylgultamic acid, low bacteria other – high isoleucine, low actinobacteria – high valine. In addition, we extracted association network consisted of high or low proportions of three types of microbes, cyanobacteria, fusobacteria, firmicutes, increased or decreased the five metabolites, carnosine, inosine, n acetlygultamic acid, deoxyinosine triphosphate, glucose.

## Discussion

### Comparison of salmoniformes with other fishes

In this study, we tried to clarify the differences between salmoniformes and other fishes to increase the usefulness of salmoniformes as farmed fish, including their relationship with intestinal microbiota, muscle metabolites, and nonnumerical information, such as habitat. We compared the pooled data (NMR [metabolite]: *n* = 1,858, NMR [collagen and lipid]: *n* = 799, NGS: *n* = 204), which were not of salmoniformes but of other fish species in our laboratory (Figures 1, 2; Supplementary Figures S1,S2,3). Here, salmoniformes had high anserine (beta-alanyl-3-methylhistidine), which has beta-alanine and 3-methylhistidine, and creatine levels; however, carnosine (beta-alanyl-L-histidine) levels were low. Both anserine and carnosine were known as antioxidants that improve human health, while creatine intake increases these two substances (Kohen et al., 1988; Derave et al., 2008; Wu, 2020). Although salmoniformes had low levels of carnosine, the stability of anserine was reported to be better than that of carnosine (Everaert et al., 2019). Therefore, the intake of salmoniformes muscle with high anserine and creatine concentrations can beneficial for human health. Salmoniformes muscle also showed high collagen and lipid levels. Since the oral intake of both macromolecules had a positive effect on the skin, it can be said that salmoniformes are excellent in these points as well. Meanwhile, the level of actinobacteria in salmoniformes was lower than that in other fishes (Supplementary Figure S2). It was reported that healthy salmoniformes had actinobacteria, while unhealthy salmoniformes had less actinobacteria in aquaculture (Wang et al., 2018). Therefore, for aquaculture, it may be necessary to consider actinobacteria for salmoniformes health care.

### Classification and association of metabolites and intestinal microbes in salmoniformes

Next, clustering was performed using i-means from NMR and NGS datasets, and important variables were extracted (Figures 3, 4). As a result, NMR 1 and 2 had higher levels of creatine than class 3 (Figure 4A). However, NMR class 1, which had the highest creatine, did not show particular association rules to other information (Figure 5B). Therefore, it could be said that the state of a fish with a high added value in creatine level in aquaculture was the one that belonged to NMR class 2 the next highest (Figure 4A). In our result, NMR class 2 was associated with nonnumeric information that the habitat was not a river but a low-flow lake or pond (Figure 5B). Therefore, to obtain high-creatine salmoniformes, it was considered desirable to cultivate them in an environment with low flow. Additionally, based on Figure 5D *O. nerka* and *O. mykiss*, which were not associated with NMR class 3, were considered useful for aquaculture. Since NGS class 3 was associated with high carnosine (Figure 5C), it would be ideal if proteobacteria and firmicutes could be increased in this environment. Finally, focusing on the relationship between the individual microbes and metabolites (Figure 6) of salmoniformes muscles, a direct association was found; an increase carnosine was associated with low levels of cyanobacteria. It is important not to leave phosphate in water for the management of cyanobacteria (Newcombe et al., 2010).

In conclusion, to extend the usefulness of salmoniformes in aquaculture, we focused on the relationship between molecular groups in muscles, intestinal microbiota, and nonnumerical information. High levels of creatine and anserine, which are beneficial to humans, were observed. Both collagen and lipid levels were also high. We propose that water quality and flow management are important using *O. nerka* and *O. mykiss* to increase their levels in an ideal aquaculture for salmoniformes. On the other hand, no correlation was found between the hardness of muscles and microbes or metabolites to control the collagen and lipid levels. Future research must be done on how these relationships contribute.

## Data availability statement

This study was carried out in compliance with the ARRIVE guidelines (http://www.nc3rs.org.uk/page.asp?id=1357).

## Ethics statement

The animal study was reviewed and approved by protocols approved by the Institutional Committee of Animal Experiments of RIKEN and adhered to the guidelines of the Institutional Regulation for Animal Experiments and Fundamental Guidelines for the Proper Conduct of Animal Experiments and Related Activities in Academic Research Institutions under the jurisdiction of the Ministry of Education, Culture, Sports, Science and Technology, Japan. No specific permission was required at any of the sampling points, as they were all in public areas.

## Author contributions

JK designed the experiments. IM, WF, KS, and DY performed experiments. HS and DY analyzed the data and created the figures. HS and JK wrote the manuscript. All authors contributed to the article and approved the submitted version.

## Funding

This work in part was supported by the grant from the Strategic Innovation Program (SIP) from Cabinet Office (CAO) of Japan.

## Conflict of interest

The authors declare that the research was conducted in the absence of any commercial or financial relationships that could be construed as a potential conflict of interest.

## Publisher’s note

All claims expressed in this article are solely those of the authors and do not necessarily represent those of their affiliated organizations, or those of the publisher, the editors and the reviewers. Any product that may be evaluated in this article, or claim that may be made by its manufacturer, is not guaranteed or endorsed by the publisher.

## Supplementary material

The Supplementary material for this article can be found online at: https://www.frontiersin.org/articles/10.3389/fmicb.2022.991819/full#supplementary-material

## Footnotes

^1^ https://cran.r-project.org/

^2^ https://gephi.org/
