# Supplementary figures and images for: Identification of salmoniformes aquaculture conditions to increase creatine and anserine levels using multiomics dataset and nonnumerical information
Source: Front Microbiol. 2022 Oct 28;13:991819. doi: 10.3389/fmicb.2022.991819 (PMC9650253; doi:10.3389/fmicb.2022.991819)

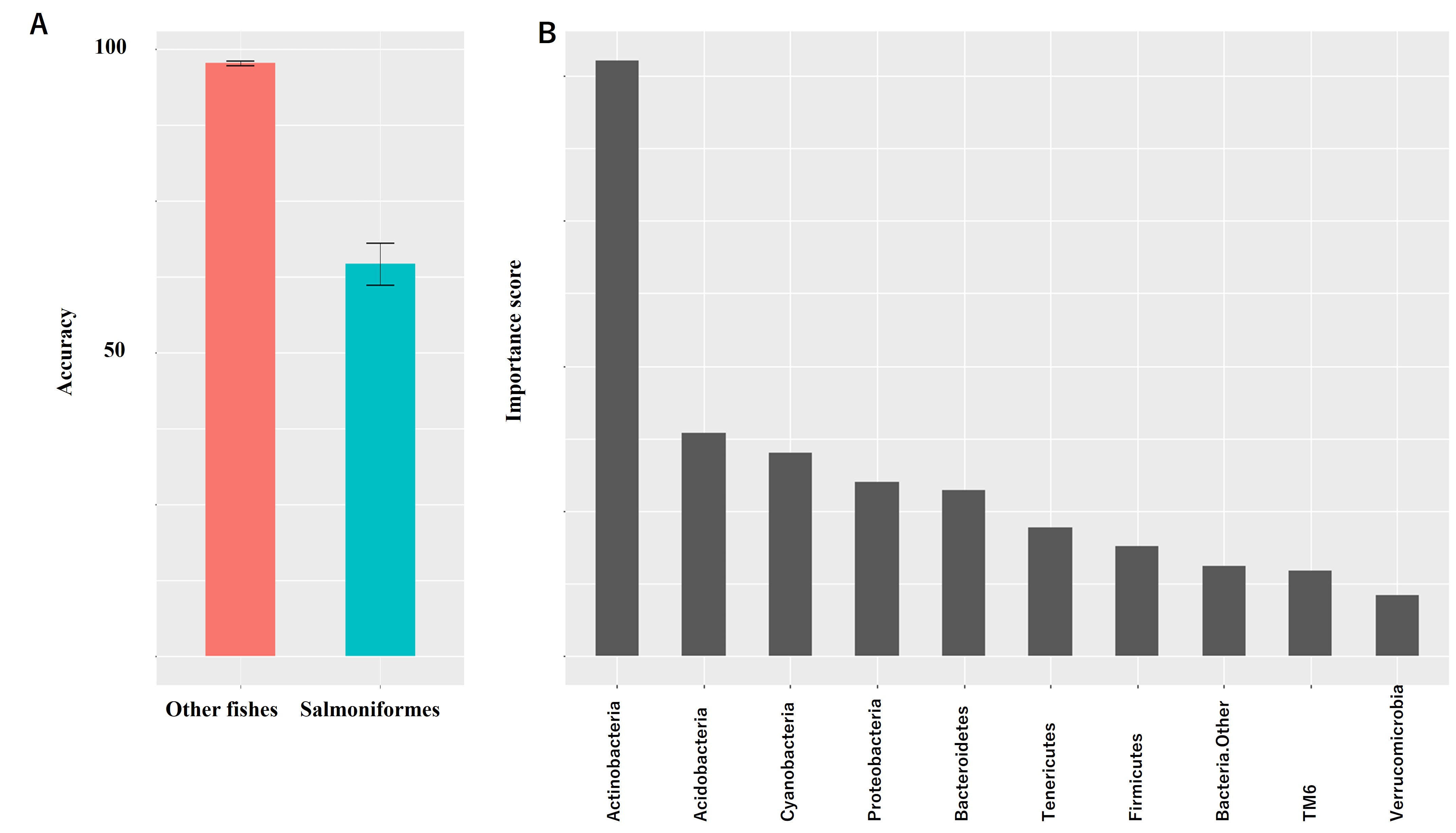

Supplement: Supplementary file 2 [file Image_1.JPEG]

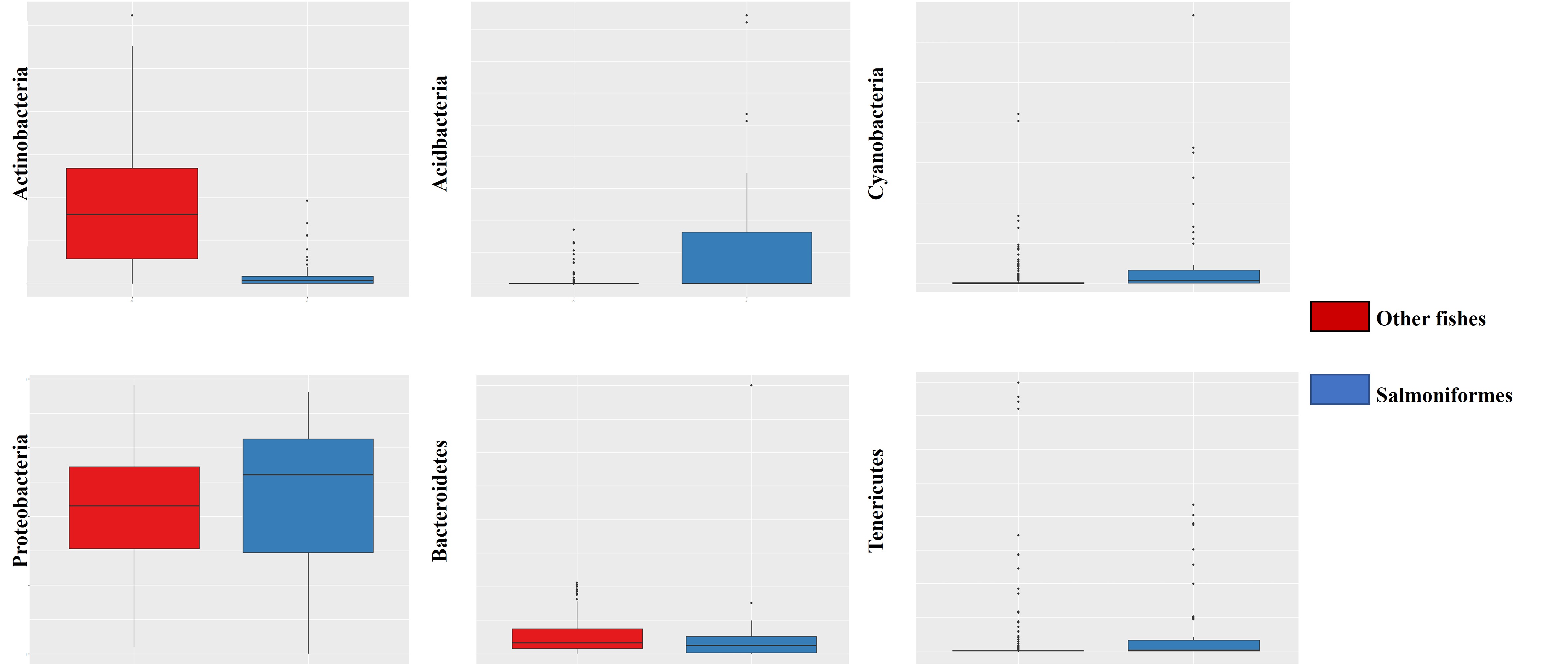

Supplement: Supplementary file 3 [file Image_2.JPEG]

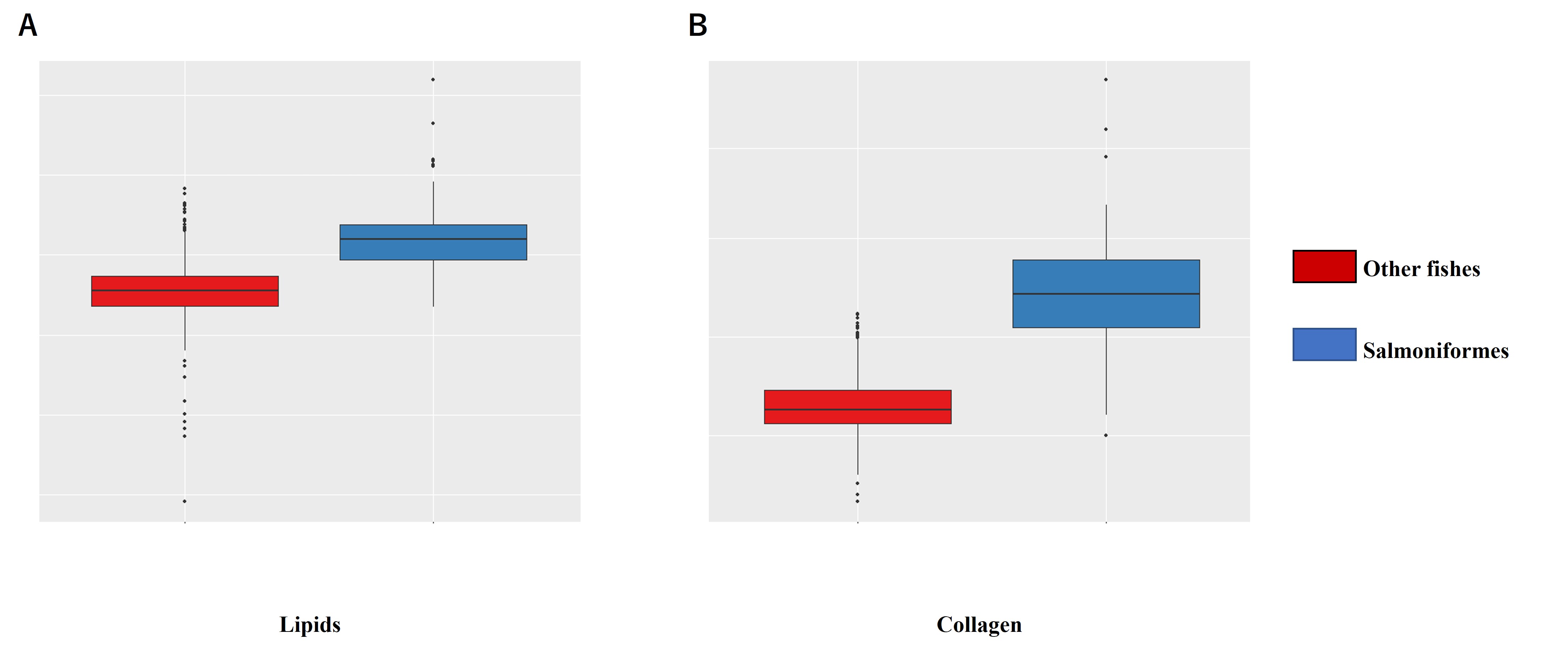

Supplement: Supplementary file 4 [file Image_3.JPEG]
